# Ketosis Suppression and Ageing (KetoSAge): The Effects of Suppressing Ketosis in Long Term Keto-Adapted Non-Athletic Females

**DOI:** 10.3390/ijms242115621

**Published:** 2023-10-26

**Authors:** Isabella D. Cooper, Yvoni Kyriakidou, Kurtis Edwards, Lucy Petagine, Thomas N. Seyfried, Tomas Duraj, Adrian Soto-Mota, Andrew Scarborough, Sandra L. Jacome, Kenneth Brookler, Valentina Borgognoni, Vanusa Novaes, Rima Al-Faour, Bradley T. Elliott

**Affiliations:** 1 Ageing Biology and Age-Related Diseases, School of Life Sciences, University of Westminster, 115 New Cavendish Street, London W1W 6UW, UK; y.kyriakidou@westminster.ac.uk (Y.K.); l.petagine@westminster.ac.uk (L.P.); andrewscarborough.research@gmail.com (A.S.); jacomenutrition@gmail.com (S.L.J.); valentina.borgognoni@gmail.com (V.B.); info@vanusanovaes.com (V.N.); rima.alfaour@gmail.com (R.A.-F.); b.elliott@westminster.ac.uk (B.T.E.); 2 Cancer Biomarkers and Mechanisms Group, School of Life Sciences, University of Westminster, London W1W 6UW, UK; k.edwards2@westminster.ac.uk; 3 Biology Department, Boston College, Chestnut Hill, MA 02467, USA; thomas.seyfried@bc.edu (T.N.S.); durajto@gmail.com (T.D.); 4 Metabolic Diseases Research Unit, National Institute of Medical Sciences and Nutrition Salvador Zubiran, Mexico City 14080, Mexico; adrian.sotom@incmnsz.mx; 5 Tecnologico de Monterrey, School of Medicine, Mexico City 14380, Mexico; 6 Retired former Research Collaborator, Aerospace Medicine and Vestibular Research Laboratory, Mayo Clinic, Scottsdale, AZ 85259, USA; kennethhbrookler@mac.com

**Keywords:** ageing, beta-hydroxybutyrate, cancer, hyperinsulinaemia, insulin resistance, ketosis, type 2 diabetes mellitus

## Abstract

Most studies on ketosis have focused on short-term effects, male athletes, or weight loss. Hereby, we studied the effects of short-term ketosis suppression in healthy women on long-standing ketosis. Ten lean (BMI 20.5 ± 1.4), metabolically healthy, pre-menopausal women (age 32.3 ± 8.9) maintaining nutritional ketosis (NK) for > 1 year (3.9 years ± 2.3) underwent three 21-day phases: nutritional ketosis (NK; P1), suppressed ketosis (SuK; P2), and returned to NK (P3). Adherence to each phase was confirmed with daily capillary D-beta-hydroxybutyrate (BHB) tests (P1 = 1.9 ± 0.7; P2 = 0.1 ± 0.1; and P3 = 1.9 ± 0.6 pmol/L). Ageing biomarkers and anthropometrics were evaluated at the end of each phase. Ketosis suppression significantly increased: insulin, 1.78-fold from 33.60 (± 8.63) to 59.80 (± 14.69) pmol/L (*p* = 0.0002); IGF1, 1.83-fold from 149.30 (± 32.96) to 273.40 (± 85.66) µg/L (*p* = 0.0045); glucose, 1.17-fold from 78.6 (± 9.5) to 92.2 (± 10.6) mg/dL (*p* = 0.0088); respiratory quotient (RQ), 1.09-fold 0.66 (± 0.05) to 0.72 (± 0.06; *p* = 0.0427); and PAI-1, 13.34 (± 6.85) to 16.69 (± 6.26) ng/mL (*p* = 0.0428). VEGF, EGF, and monocyte chemotactic protein also significantly increased, indicating a pro-inflammatory shift. Sustained ketosis showed no adverse health effects, and may mitigate hyperinsulinemia without impairing metabolic flexibility in metabolically healthy women.

## 1. Introduction

The 21st century bears the hallmark of an ageing global population, in an estimated 8 billion people by 2023 [1]. By 2030, one in every six Europeans are expected to be aged over 60 years, and by 2040, a quarter of older adults will surpass 85 years of age [2]. This demographic shift gains paramount significance when viewed through the prism of health implications associated with ageing. In 2019 and 2022, the leading cause of death for females in England and Wales was Alzheimer’s disease (AD) and other dementias [3], followed by cardiovascular disease (CVD) and stroke, as well as cancers including: tracheae, bronchus, and lung; colon and rectum; prostate; breast; and lymphomas and multiple myeloma [4]. These diseases also top the leading causes of death in the United States, with CVD leading, closely followed by AD and cancers (WHO, 2019). Analysed data of 8721 participants from the National Health and Nutrition Examination Survey 2009–2016 showed that the proportion of metabolically healthy Americans decreased from 19.9% to 12.2%, which means 87.8% were metabolically unhealthy and on the hyperinsulinaemia spectrum [5,6]. Ageing is associated with increased risk and rates of non-communicable chronic diseases, including CVD, AD, hypertension, type 2 diabetes mellitus (T2DM), metabolic syndrome (MetS), non-alcoholic fatty liver disease (NAFLD), chronic inflammation, and cancer [6]. These conditions detrimentally affect quality of life, healthspan, and lifespan. Specifically, MetS emerges as a direct consequence of chronic hyperinsulinaemia, which is closely linked to inflammation [6,7,8,9].

Lifespan, healthspan, and cellular health are greatly influenced by nutrient availability. When the availability of nutrients is low, cells prioritise essential functions over cell division, which slows progression through their replicative cycles preserving their Hayflick limit [10]. Carbohydrate restriction (CR) and fasting have been shown to extend lifespan [11]. Cells are capable of sensing energy availability and nutrient types, activating intracellular signalling pathways to stimulate anabolic or catabolic processes which affect cellular health and longevity [12,13,14]. Glucose, fatty acids, D-beta-hydroxybutyrate (BHB), and protein metabolic substrates serve as indicators of the body’s overall metabolic condition and nutrient availability. CR and fasting induce a metabolic phenotype called ketosis [8], characterised by decreased glucose and insulin levels and elevated BHB concentration; this state is termed nutritional ketosis (NK) when detectable plasma BHB concentration begins to exceed > 0.3 mmol/L and < 10 mmol/L, with endogenous insulin production below a level that inhibits ketogenesis [8].

Chronic insulin secretion and signalling, driven by dietary sources of glucose, leads to hyperinsulinaemia and/or insulin resistance, and consequently chronic diseases which decrease healthspan by accelerating cellular growth and division whilst impeding apoptosis and promoting production of inflammatory cytokines. Reducing insulin and insulin-like growth factor receptor signalling (IIS) as well as increasing BHB has been found to increase lifespan and healthspan in model organisms and animal studies [11,15,16,17]. Conversely, ketosis has been shown to increase healthspan and lifespan through mechanisms such as promoting transcription of longevity-related genes, increasing autophagy, mitophagy, and mitochondrial biogenesis, and enhancing antioxidant production [6,17,18,19,20]. Fasting mimicking diets (FMD), including ketogenic diets, upregulate beta-oxidation, ketogenesis and ketolysis, enhance mitophagy, increase mitochondrial biogenesis, and alter gene expression, promoting oxidative stress responses and cell survival [6,21,22,23,24].

Historical and emerging research demonstrates the positive impact of ketogenic metabolic therapy (KMT) in treating and preventing neurological diseases, CVD, cancer, T2DM, and chronic inflammation [25]. Insulin negatively regulates 3-hydroxy-3-methylglutaryl-COA (HMG-CoA) synthase, the rate-limiting enzyme for ketogenesis [19,26]. Dietary farinaceous and sucrose-rich foods are potent stimulators of bolus insulin secretion [8,27]. Repeated bolus glucose excursions chronically stimulate bolus insulin synthesis and release, and over time downregulate ketogenesis enzyme expression, leading to chronic hypoketonaemia [8,26,28]. There are a paucity of trials studying long-standing ketosis metabolically healthy individuals who sustain ketosis as their normal metabolic phenotype 1 lifestyle [8], and even fewer on active, yet non-athletic females. We therefore studied the effect of suppressing ketosis for 21 days in this demographic cohort. In order to suppress ketosis, participants followed the Standard U.K. (SUK) dietary guidelines, which recommend the daily consumption of at least 267 g of carbohydrate per day for women [29]. Following the intervention to suppress ketosis, participants returned to NK and were reassessed 21 days later to better understand if changes seen after suppression of ketosis for 21 days were due to the intervention, and to investigate metabolic flexibility.

## 2. Results

### 2.1. Suppression of Ketosis Increases BMI and Fat Mass

Following 21-days suppression of ketosis (SuK), phase 2 (P2), there were significant increases in both weight from baseline NK, phase 1 (P1) 52.99 kg (± 4.24) to 55.65 kg (± 4.10, P2; *p* = 0.0002), and BMI, from 20.52 (± 1.39, P1) to 21.54 (± 1.30, P2; *p* < 0.0001), in all participants, compared to NK; P1 (Table 1). Fat mass and TBW also increased from 14.21 kg (± 2.55, P1) to 15.88 kg (± 2.23, P2; *p* = 0.0008) and from 28.15 L (± 2.87, P1) to 29.15 L (± 2.96, P2; *p* = 0.0016), respectively (Table 1). Additionally, both waist-to-hip and waist-to-height ratios increased significantly in P2 compared to P1 (Table 1).

These trends then reversed after the removal of SuK at the end of 21-days, reverting to NK, phase 3 (P3), compared to P2. Both weight (53.93 kg ± 4.04; *p* < 0.0001, P3) and BMI (20.82 ± 1.46; *p* = 0.0025, P3) trended back towards baseline in all but one participant, where only a small increase in weight was observed (+0.2 kg). Concordantly, the decreases in weight between the phases were accompanied by significant decreases in fat mass (14.78 kg ± 2.20; *p* = 0.0057, P3) and TBW (28.42 L ± 3.15; *p* = 0.0026, P3). These changes were also accompanied by decreases in both waist-to-hip and waist-to-height ratios (Table 1).

**Figure 1:**
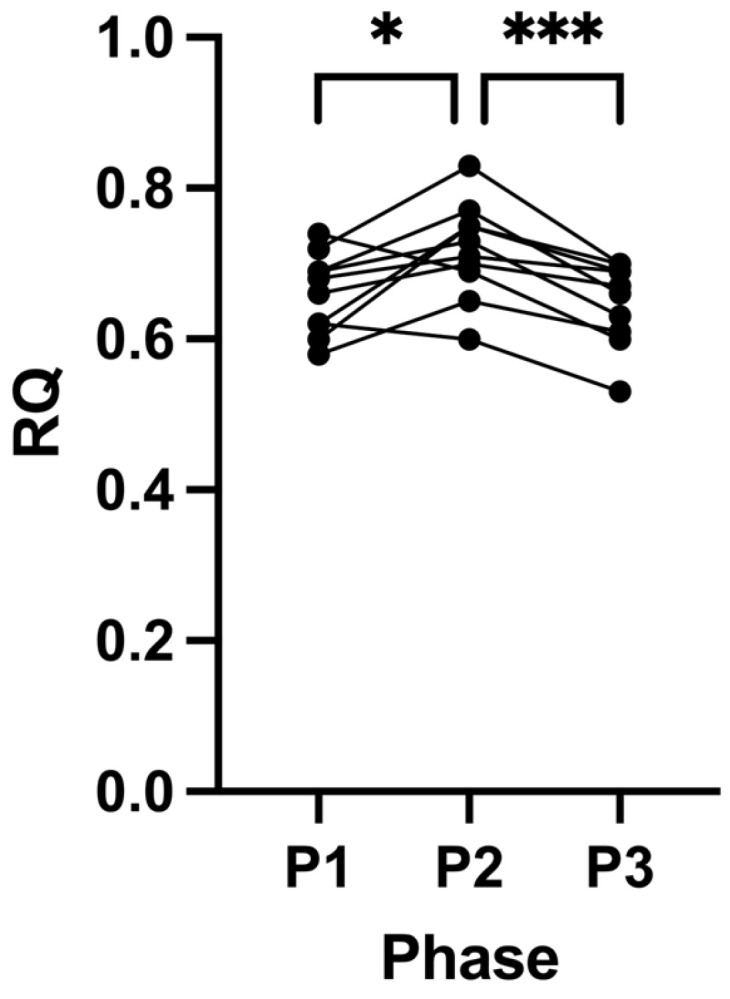
Respiratory quotient (RQ) measurements across all phases. Measurements were taken following each of the study phases: baseline nutritional ketosis (NK) P1; intervention to suppress ketosis (SuK) P2; and removal of SuK returning to NK P3; RQ was determined by indirect calorimetry. Measurements were taken at 8 a.m. after a 12 h overnight fast; (n = 10); * *p* < 0.05; *** *p* < 0.001.

RQ increased in most participants (80%) following P2 (0.66 ± 0.05, P1 to 0.72 ± 0.06, P2; *p* = 0.0427; Figure 1). After removal of the intervention at the end of P3, we observed a decrease in RQ in all participants, returning to their baseline (0.65 ± 0.06, P3; *p* = 0.0005; Figure 1). There were no changes in either systolic or diastolic blood pressure across all the study phases (Table 1).

### 2.2. Adherence

Based on the study protocol, participants were required to self-report 252 capillary BHB concentrations: 84 tests across each of the phases (Figure 7 in Section 6). The number of fulfilled tests and percentage of completed tests out of the possible 252 for all participants is shown in Table 2. The average percentage of successful tests was 99.37%, with four participants completing 100% of all 252 potential tests.

The mean capillary BHB concentration significantly decreased from 1.9 mmol/L (± 0.7) in the baseline ketosis phase (P1) to 0.1 mmol/L (± 0.1) following the suppression of ketosis phase (P2; *p* < 0.0001). During P3, mean capillary BHB concentration increased significantly (*p* < 0.0001) and returned to baseline (1.9 ± 0.6 mmol/L). The maintenance of high mean capillary BHB concentrations (> 0.5 mmol/L) during P1 and P3 indicated that all participants adhered to the requirements to maintain ketosis during these phases. Similarly, the low levels of BHB during P2 indicated adherence to the study protocol, whereby participants effectively suppressed nutritional ketosis (Table 2).

There were variations in capillary BHB concentrations across the daily tests. The frequencies of tests which satisfied different cut-offs are summarised in Table 3. During P1 and P3, almost all reported capillary BHB concentrations were > 0.3 mmol/L or ≥ 0.5 mmol/L, which are generally considered the cut-off for ketosis or nutritional ketosis, respectively [8,30]. There were very few tests meeting these thresholds in P2, compared to P1 and P3 (Table 3).

During P1, 2/252 tests for two participants showed capillary BHB concentrations of 0.3 mmol/L, and 8/252 and 2/252 for the same two participants in P2 and P3, respectively (Table 2). There were no reported capillary BHB concentrations of < 0.1 mmol/L in either P1 or P3 (Table 3).

During P2, the majority of capillary BHB concentrations were < 0.3 mmol/L, with a significant proportion of readings < 0.1 mmol/L. There were also some instances of capillary BHB readings > 0.3 mmol/L or ≥0.5 mmol/L during P2 (Table 3). These tests were often early in the morning after an overnight fast and during the first days of SuK.

The high level of adherence to testing, coupled with the expected high concentrations of capillary BHB in P1 and P3, and low concentrations of capillary BHB in P2, indicated high levels of adherence to the protocol throughout the entire study.

### 2.3. Suppression of Ketosis Is Associated with Increases in Insulin, IGF-1, Glucose and T3

**Figure 2:**
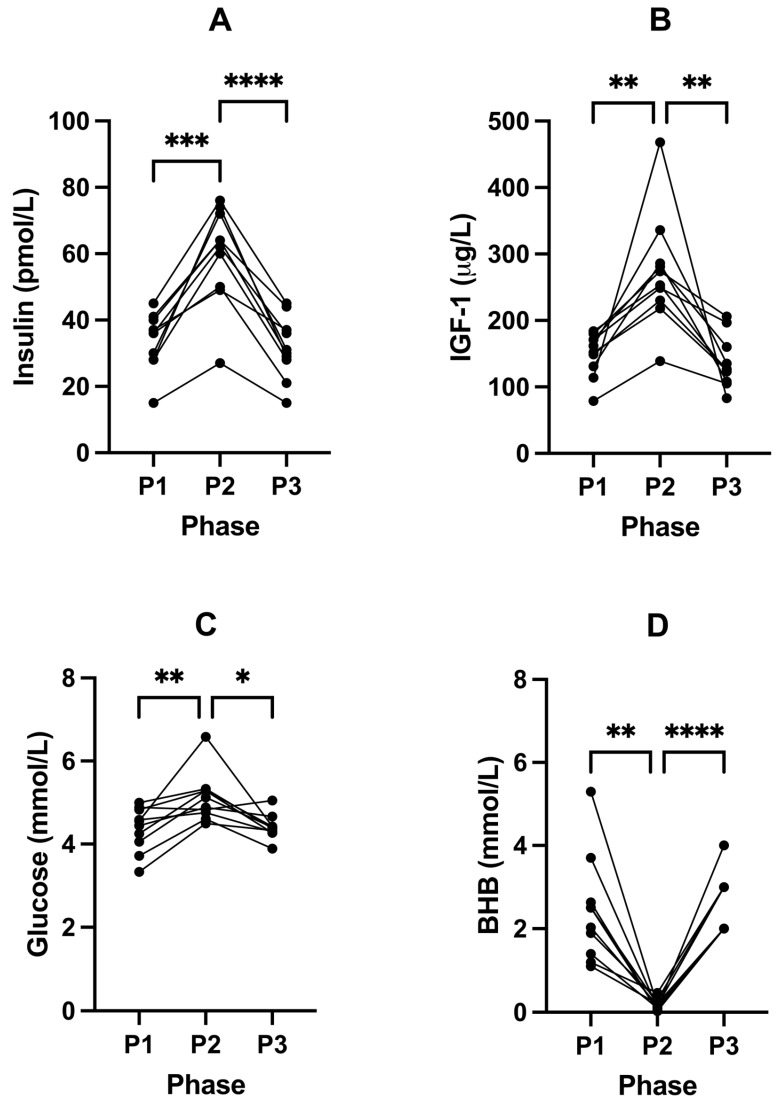
Changes in fasted blood insulin (**A**), IGF-1 (**B**), glucose (**C**), and BHB (**D**) concentrations across all phases. Measurements were taken following each of the study phases: baseline nutritional ketosis (NK) P1; intervention to suppress ketosis (SuK) P2; and removal of SuK returning to NK, P3. Measurements were taken at 8 a.m. after a 12 h overnight fast; (n = 10). * *p* < 0.05; ** *p* < 0.01; *** p < 0.001; **** p < 0.0001.

**Figure 3:**
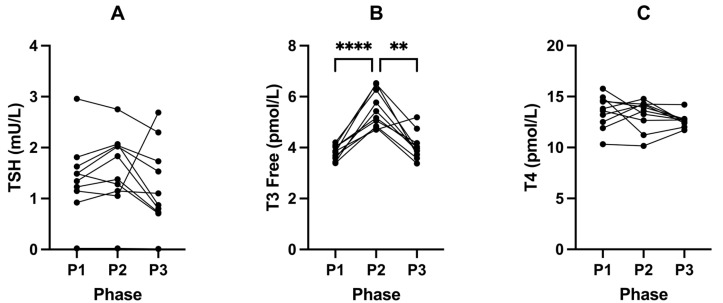
Changes in TSH (**A**), T3 Free (**B**), and T4 (**C**) concentrations across all phases. Measurements were taken following each of the study phases: baseline nutritional ketosis (NK) P1; intervention to suppress ketosis (SuK) P2; and removal of SuK returning to NK P3. Measurements were taken at 8 a.m. after a 12 h overnight fast; (n = 10). ** *p* < 0.01; **** *p* < 0.0001.

Following P2, fasting insulin significantly increased from 33.60 pmol/L (± 8.63, P1) to 59.80 pmol/L (± 14.69, P2; *p* = 0.0002; Figure 2A) and IGF-1 from 149.30 µg/L (± 32.96, P1) to 273.40 µg/L (± 85.66, P2; *p* = 0.0045; Figure 2B) compared to P1 (Table 4). This was accompanied by a significant increase in blood glucose from 4.36 (± 0.53) to 5.12 mmol/L (± 0.59, P2; *p* = 0.0088) (in mg/dL: 78.6 (± 9.5) to 92.3 (± 10.6)); Figure 2C) and decrease in BHB concentrations from 2.43 (± 1.28) to 0.18 mmol/L (± 0.13, P2; *p* = 0.0012); Figure 2D; Table 4). Free T3 also significantly increased from 3.81 pmol/L (± 0.28, P1) to 5.51 pmol/L (± 0.72, P2; *p* = < 0.0001; Figure 3B) following P2.

These trends reversed following P3, where we observed significant changes in the concentrations of insulin (*p* < 0.0001; Figure 2A), IGF-1 (*p* = 0.0055; Figure 2B), glucose (*p* = 0.0177; Figure 2B), BHB (*p* < 0.0001; Figure 2D), and free T3 (*p* = 0.0015; Figure 3B; Table 4), compared to P2.

### 2.4. Oral Glucose Tolerance Tests

#### 2.4.1. Between Phases (P1 vs. P2 vs. P3) OGTT Glucose Response

**Figure 4:**
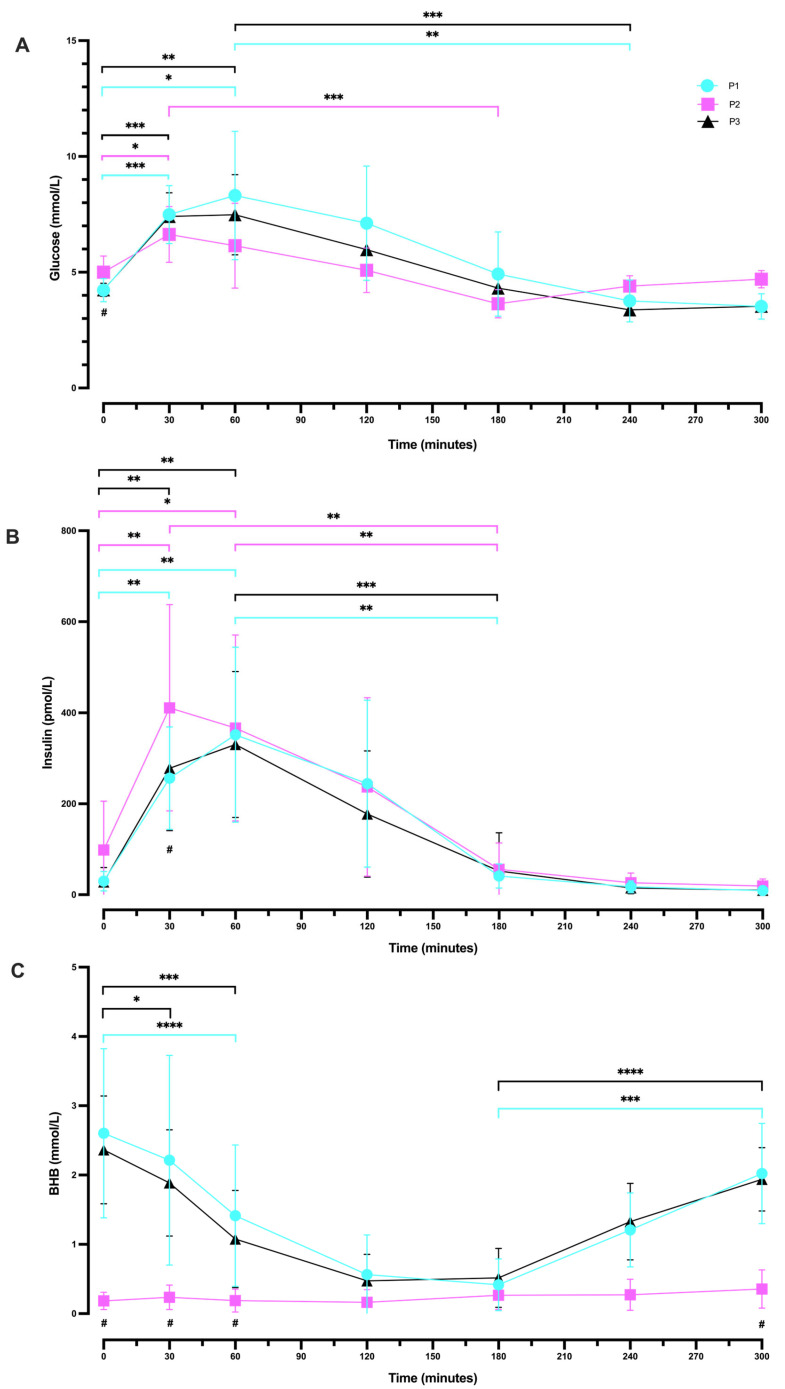
Differences in glucose (**A**), insulin (**B**), and BHB (**C**) in response to oral glucose tolerance tests across all study participants in all phases. Measurements were taken following each of the study phases: baseline nutritional ketosis (NK) P1 (blue circles); intervention to suppress ketosis (SuK) P2 (pink squares); and removal of SuK returning to NK, P3 (black triangles); (n = 10). The connected line indicates group means (± SD); * indicates significant difference within each phase across different time points; # indicates significant difference between phases at the time point as indicated. * p < 0.05; ** p < 0.01; *** p < 0.001; **** p < 0.0001.

Basal values of blood glucose measured as part of the OGTT were lower in P1 and P3 (4.23 mmol/L ± 0.50 and 4.24 mmol/L ± 0.28, respectively) compared to P2 (5.01 mmol/L ± 0.70; Figure 4A). We found significant differences in mean glucose concentration amongst the three phases at baseline (*p* = 0.0016). Post hoc comparisons showed a significant difference in glucose concentration between P1 and P2 (*p* = 0.0065) and between P2 and P3 (*p* = 0.0166).

P1 and P3 showed a similar pattern in blood glucose response, whereby glucose peaked at 60 min (8.31 mmol/L ± 2.78 and 7.48 ± 1.73, respectively; *p* = 0.4385). However, in P2, glucose concentration reached a peak earlier, at 30 min (6.63 mmol/L ± 1.20). P1 and P3 showed a trough in glucose response at 240 min (3.76 mmol/L ± 0.91 and 3.38 mmol/L ± 0.20, respectively; *p* = 0.4137), whereas glucose concentration in P2 dropped earlier at 180 min (3.64 mmol/L ± 0.61). In P1 and 3, by minutes 240 and 300, glucose continued to trend down, whereas in P2, glucose trends up at these time points. By 300 min, values returned to their phase baselines.

#### 2.4.2. Within-Phase Glucose Response during a 5 h OGTT

There were significant changes in glucose concentration across seven time points (0, 30, 60, 120, 180, 240, and 300 min) in P1 (*p* < 0.0001; Figure 4A). More specifically, there was a statistically significant increase in blood glucose change from 0 min (4.23 mmol/L ± 0.50) to 30 min (7.49 mmol/L ± 1.25; *p* = 0.0009) and to 60 min (8.31 mmol/L ± 2.78; *p* = 0.0287). There was also a significant difference between the peak of glucose at 60 min (8.31 mmol/L ± 2.78) and at 240 min (3.76 mmol/L ± 0.91; *p* = 0.0070), which continued to decrease at 300 min (3.52 mmol/L ± 0.55; *p* = 0.0068).

Following P2, there were significant changes in glucose concentration amongst all time points (*p* = 0.0002; Figure 4A). More precisely, glucose concentration was significantly increased from 0 min (5.01 mmol/L ± 0.70) to 30 min (6.63 mmol/L ± 1.20; *p* = 0.0435), but not between 0 and 60 min. There were also statistically significant differences between the peak at 30 min (6.63 mmol/L ± 1.20) and the trough at 180 min (3.64 mmol/L ± 0.61; *p* = 0.0002).

After returning to P3, significant changes were observed in glucose concentration amongst all time points (*p* < 0.0001; Figure 4A). There was a statistically significant increase from 0 min (4.24 mmol/L ± 0.28) to 30 min (7.41 mmol/L ± 1.02; *p* = 0.0001) and to 60 min (7.48 mmol/L ± 1.73; *p* = 0.0059). Similarly, to P1, there was also a significant difference between the peak of glucose at 60 min (7.48 mmol/L ± 1.73) and the trough at 240 min (3.38 mmol/L ± 0.20; *p* = 0.0004).

#### 2.4.3. Following Plateau, Blood Glucose Concentration Increased during Ketosis Suppression

After 180 min in P2, glucose began to trend upwards, which was not observed in P1 or P3. At 240 min, whilst glucose concentration was higher in P2 (4.40 mmol/L ± 0.45) vs. P1 (3.76 mmol/L ± 0.90), this difference was not statistically significant (*p* = 0.1342). However, glucose concentration was significantly higher in P2 (4.40 mmol/L ± 0.45) compared to P3 (3.38 ± 0.20; *p* = 0.0007). Similarly, at 300 min glucose concentration was significantly higher in P2 (4.70 mmol/L ± 0.38) compared to P1 (3.52 ± 0.55; *p* = 0.0001) and to P3 (3.53 ± 0.13; *p* < 0.0001). There was no difference in the concentration of glucose at 240 min (*p* = 0.4137) or at 300 min (*p* = 0.9988) when comparing P1 and P3; Figure 4A.

#### 2.4.4. Between Phases (P1 vs. P2 vs. P3) OGTT Insulin Response

Fasting insulin concentrations were also found to be lower in P1 and P3 (29.94 pmol/L ± 21.48 and 27.97 pmol/L ± 31.68, respectively) in comparison to P2 (98.19 pmol/L ± 107.69), although they were not significantly different (P1 vs. P2; *p* = 0.0971, and P2 vs. P3, *p* = 0.0754; Figure 4B). Insulin concentration peaked at 30 min in P2 (411.07 pmol/L ± 226.59) and at 60 min in P1 and P3 (351.94 pmol/L ± 192.36 and 330.27 pmol/L ± 160.56, respectively). However, insulin concentration returned to baseline values in all phases with a similar pattern at the end of the experimental period (300 min). We also analysed changes in insulin concentration at 30 min amongst the three phases. The data showed a significant difference between P1 (256.27 pmol/L ± 112.59) and P2 (411.07 pmol/L ± 226.59; *p* = 0.0324), and between P2 (411.07 pmol/L ± 226.59) and P3 (278.23 pmol/L ± 137.20; *p* = 0.0161).

#### 2.4.5. Within-Phase Insulin Response during a 5 h OGTT

Following P1, repeated measures one-way ANOVA illustrated statistically significant changes in insulin concentration across all time points (*p* < 0.0001; Figure 4B). More precisely, there was a significant increase from 0 min (29.94 pmol/L ± 21.48) to 30 min (256.27 pmol/L ± 112.59; *p* = 0.0013) and to 60 min (351.94 pmol/L ± 192.36; *p* = 0.0047). There was also a significant difference between the peak at 60 min (351.94 pmol/L ± 192.36) and 180 min (41.08 pmol/L ± 26.62; *p* = 0.0069).

P2 also showed significant changes in insulin concentrations amongst all time points (*p* < 0.0001; Figure 4B). More specifically, there was a statistically significant increase from 0 min (98.19 pmol/L ± 107.69) to 30 min (411.07 pmol/L ± 226.59; *p* = 0.0011) and to 60 min (366.68 pmol/L ± 204.21; *p* = 0.0291). Additionally, there was a statistically significant difference between the peak at 30 min (411.07 pmol/L ± 226.59) and 180 min (55.95 pmol/L ± 57.99; *p* = 0.0054), and between that at 60 min (366.68 pmol/L ± 204.21) and 180 min (55.95 pmol/L ± 57.99; *p* = 0.0028).

Similarly, after returning to P3, insulin concentrations significantly changed across all time points (*p* < 0.0001; Figure 4B). Insulin levels were significantly increased from 0 min (27.97 pmol/L ± 31.68) to 30 min (278.23 pmol/L ± 137.20; *p* = 0.0024) and to 60 min (330.27 pmol/L ± 160.56; *p* = 0.0015). As in P1, there was a significant difference between the peak at 60 min (330.27 pmol/L ± 160.56) and 180 min (52.11 pmol/L ± 84.10; *p* = 0.0005).

Notably, in all three phases, insulin concentrations began to converge and trend significantly downwards at 180 min. At 240 and 300 min, the concentration of insulin began to plateau.

#### 2.4.6. Between Phases (P1 vs. P2 vs. P3) OGTT BHB Response

Basal values of BHB were higher in P1 and P3 (2.60 mmol/L ± 1.22 and 2.36 mmol/L ± 0.78, respectively) than in P2 (0.18 mmol/L ± 0.12), and they significantly differed between P1 vs. P2 (*p* = 0.0004) and between P2 vs. P3 (*p* < 0.0001; Figure 4C).

A statistically significant difference was also observed amongst the three phases in mean BHB concentration at 30 min (*p* = 0.0020), at 60 min (*p* = 0.0034), and at 300 min (*p* < 0.0001). Post hoc testing indicated that BHB concentration significantly differed between P1 (2.22 mmol/L ± 1.51) and P2 (0.24 mmol/L ± 0.18; *p* = 0.0078) and between P2 (0.24 mmol/L ± 0.18) and P3 (1.89 mmol/L ± 0.77; *p* = 0.0004) at 30 min. In addition, BHB concentration at 60 min was significantly different between P1 (1.41 mmol/L ± 1.02) and P2 (0.19 mmol/L ± 0.17; *p* = 0.0107), and between P2 (0.19 mmol/L ± 0.17) and P3 (1.08 mmol/L ± 0.70; *p* = 0.0039). Further, there were significant differences between P1 (2.02 mmol/L ± 0.72) and P2 (0.36 mmol/L ± 0.28; *p* < 0.0001) and between P2 (0.36 mmol/L ± 0.28) and P3 (1.94 mmol/L ± 0.46; *p* < 0.0001) at 300 min.

#### 2.4.7. Within-Phase BHB Response during a 5 h OGTT

There were statistically significant changes in BHB concentration across time points overall in P1 (*p* = 0.0006; Figure 4C), with no significant changes between 0 min (2.60 mmol/L ± 1.22) and 30 min (2.22 mmol/L ± 1.51; *p* = 0.2056). However, there was a significant decrease from 0 min (2.60 mmol/L ± 1.22) to 60 min (1.41 mmol/L ± 1.02; *p* < 0.0001). Whilst there were minimal changes in BHB concentration across all time points following P2 (*p* = 0.0961; Figure 4C), after returning to P3, there were significant changes across time points (*p* < 0.0001). More specifically, there was a significant decrease from 0 min (2.36 mmol/L ± 0.78) to 30 min (1.89 mmol/L ± 0.77; *p* = 0.0444), and to 60 min (1.08 mmol/L ± 0.70; *p* = 0.0001).

Following the 75 g glucose loaded drink, both P1 and P3 demonstrated a similar pattern response in BHB change, with BHB concentration showing a steady time-dependent decrease until 120 min. Subsequently returning in a linear recovery from 180 min (0.42 mmol/L ± 0.37, P1; 0.52 mmol/L ± 0.42, P3), with a significant increase until 300 min (2.02 mmol/L ± 0.72; *p* = 0.0005, P1; 1.94 mmol/L ± 0.46; *p* < 0.0001, P3). Conversely, during SuK (P2), BHB concentration followed a similar response across time, whereby there were minimal changes throughout the experimental period (Figure 4C).

### 2.5. Suppression of Ketosis Is Associated with Increases in Inflammatory Liver Markers

**Figure 5:**
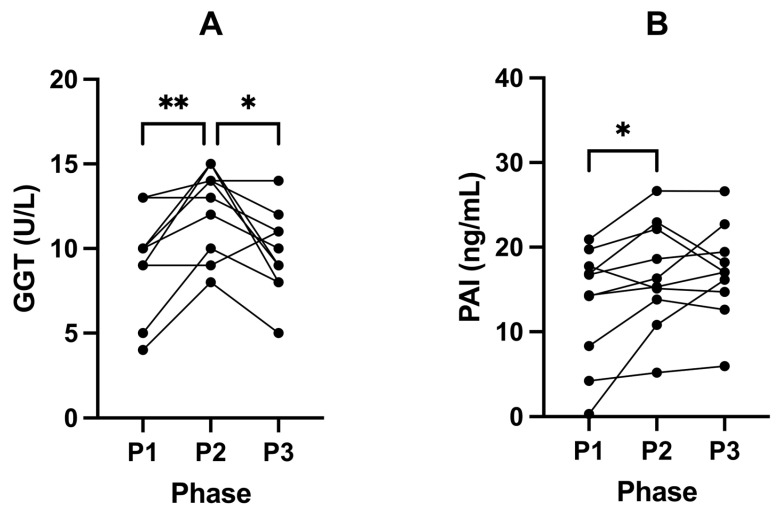
Changes in liver parameters GGT (**A**) and PAI-1 (**B**) in participants across all phases. Measurements were taken following each of the study phases: baseline nutritional ketosis (NK) P1; intervention to suppress ketosis (SuK) P2; and removal of SuK returning to NK, P3. Measurements were taken at 8 a.m. after a 12 h overnight fast; (n = 10). * *p* < 0.05; ** *p* < 0.01.

Following P2, GGT concentrations increased significantly in all participants from 9.60 U/L (± 3.13) in P1 to 12.40 U/L (± 2.55) in P2 (*p* = 0.0087; Figure 5A). From P2 to P3, GGT levels were significantly reduced to 9.70 U/L (± 2.50; *p* = 0.0286; Figure 5A; Table 5). We also found that SuK (P2) significantly increased PAI-1 levels from 13.34 ng/mL (± 6.85, P1) to 16.69 ng/mL (± 6.26, P2; *p* = 0.0428). No changes in PAI-1 levels were observed following P3 (17.05 ng/mL ± 5.58) compared to P2 (*p* = 0.9483; Figure 5B; Table 5).

CRP was found to be low or less than the lowest detectable limit of the assay in all participants across all study phases. CRP was therefore measured using a high-sensitivity assay (ultra-sensitive CRP) in five participants (Table 5). Despite this, no significant changes were determined from P1 (1.00 mg/L ± 1.19) to P2 (1.16 mg/L ± 1.56; *p* = 0.9938); or from P2 to P3 (1.35 mg/L ± 2.23; *p* = 0.7477). We found no statistically significant changes in all other liver or lipid markers across all phases of the study (Table 5).

### 2.6. Ketosis Maintains Lower Levels of EGF, VEGF and MCP-1

**Figure 6:**
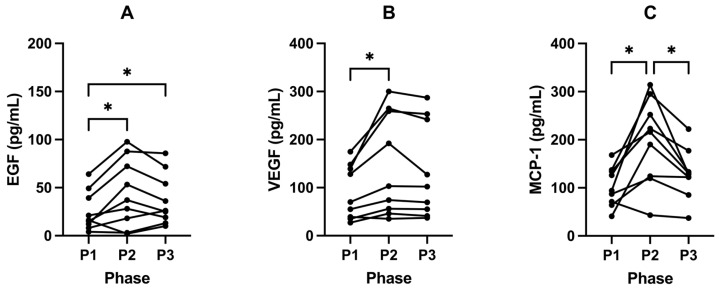
Fasted EGF (**A**), VEGF (**B**) and MCP-1 (**C**) in participants across all phases. Measurements were taken following each of the study phases: baseline nutritional ketosis (NK) P1; intervention to suppress ketosis (SuK) P2; and removal of SuK returning to NK, P3. Measurements were taken at 8 a.m. after a 12 h overnight fast; *(A and B, n = 10; C, n = 9)*. * *p* < 0.05.

There were increases in EGF from 33.02 pg/mL (± 30.96) in P1 to 50.13 pg/mL (± 38.19) following P2 (*p* = 0.0450; Figure 6A; Table 6). VEGF also increased from 93.93 pg/mL (± 54.30) in P1 to 147.33 pg/mL (± 100.03) following P2 (*p* = 0.0314; Figure 6B; Table 6). MCP—1 significantly increased from 103.98 pg/mL (± 39.30) in P1 to 192.53 (± 84.73) following P2 (*p* = 0.0137; Figure 6C; Table 6).

Following P3, these growth factors and cytokines trended back to baseline and decreased significantly compared to P2. EGF (*p* = 0.3473; Figure 6A; Table 6) and VEGF (*p* = 0.2102; Figure 6B; Table 6) decreased to 37.82 pg/mL (± 26.81) and 134.80 pg/mL (± 98.79), respectively. Concentrations of MCP-1 also decreased significantly to 128.52 pg/mL (± 51.80) following P3 compared to P2 (*p* = 0.0175; Figure 6C; Table 6).

There were minimal changes in IL-1b following P2 (*p* = 0.7045). However, following P3, all participants had a significantly decreased expression of IL-1b (*p* = 0.0381; Table 6). Similarly, there were minimal changes following P2 in the expression of TNF-α (*p* = 0.3887); however, in P3, 86% of participants showed a decreased expression of TNF—α (*p* = 0.0785; Table 6). We found no change in all other cytokines and growth factors across all phases of the study (Table 6).

## 3. Discussion

There have been many studies investigating the effect of ketosis in humans; however, little is known about the physiological adaptations in individuals who have never had a metabolic illness and maintained long-term (>1 year) habitual ketosis for more than 80% of their year. Furthermore, prior work has primarily examined males. Our cohort self-reported to have sustained nutritional ketosis for an average of 3.9 years, with confirmed NK for at least 6 months in the lead-in period to the trial. Participants presented with healthy weights, BMI, waist-to-hip, waist-to-height, and blood pressure (Table 3). Molecular markers including lipid panels, liver enzymes (Table 5), and cytokines (Table 6) were also within healthy ranges. Thus, our data indicate that long term NK, euketonaemia, does not have a negative effect on health in this cohort.

Euketonaemia is defined as a state of ketosis that is not associated with any harmful effects. Although, in most cases, the term has been used to refer to a state of normal ketonemia in patients with diabetes, the term has also been used in a broader sense to refer to a state of normal ketonemia in healthy individuals [31,32]. Euketonaemia in adults has been associated with improved insulin sensitivity and euglycaemia [33,34,35]. Moreover, euketonaemia is associated with reduced inflammation in the brain [36,37], is consistent with evolutionary biology, and has a protective effect on mitochondria [38].

### 3.1. Macroscopic Changes/Anthropometrics

Throughout the intervention phase (P2), where participants were actively suppressing ketosis, participants often reported capillary BHB concentrations of > 0.3 mmol/L after the overnight fast, and even three hours after a carbohydrate-containing meal (Table 2). Together, these data indicate that the participants were indeed highly fat-adapted and, even with the introduction of carbohydrates into their diet, their bodies reverted to beta-oxidation and ketolysis during periods of fasting. Following P3, participants tracked back to the baseline level of ketosis as indicated by their capillary BHB levels (Figure 2D). The participants enrolled in this study were able to tolerate 21 days of suppression of ketosis and the consequent upregulation of glucose metabolism and still return to their baseline level of ketosis. This suggests that metabolic flexibility is maintained in long-term habitual ketosis in metabolically healthy individuals.

Across all three phases, in two different metabolic states (ketosis vs. glucose fuelling), participants’ RQ values were indicative of individuals that were metabolically healthy; interestingly, their values were superior to observations previously made in high performance athletes [39,40,41,42]. Even after 21 days of SuK, a 12 h overnight fast still induced higher fat oxidation, as evidenced by their P2 RQ values (Figure 1; Table 1). However, surprisingly, there was still a significant difference between P2 compared to P1 and P3, even within the overall highly fat-adapted state that all the participants were in, in all three phases after an overnight fast (Figure 1). Given that the baseline RQ values were indicative of a high state of beta-oxidation, it was not expected that a 21-day of SuK would result in RQ measurements that were statistically inferior to those observed at baseline (P1).

Three weeks of SuK resulted in changes in body composition, with increases in weight and BMI. This was largely accounted for by increases in TBW and total body fat (Table 1). However, the increased body composition measurements taken following P2 (and indeed, P1 and P3) were still within normal ranges [43,44] and tracked back to baseline levels following P3.

### 3.2. Insulin, IGF-1, and Glucose

In the normative setting, the most influential pancreatic insulin secretagogue is dietary carbohydrate, whilst basal insulin release is regulated by a multitude of factors including hepatic glycogenolysis, which is further regulated by glucagon, osteocalcin, and other secretagogues [8]. It is interesting that with the increased repeated stimulation of bolus insulin during P2, fasting (basal) insulin and glucose subsequently increased (Figure 2). This is likely due to insulin’s systemic effects, where enforced glucose fuelling results in increased glucose demand. In addition, insulin’s suppressive effect on beta-oxidation, where neither lipid provision for beta-oxidation nor ketogenesis is sufficient due to insulin also inhibiting insulin sensitive lipase, which is required to release lipids from adipocytes [45,46,47]. Therefore, the upregulation of hepatic glycogenolysis occurs in response to chronic insulin signalling.

Participants’ habitual ketosis lifestyle also demonstrated significantly lower IGF-1 levels in P1 and P3 (Figure 3). IGF-1 is regulated by insulin on multiple fronts; regulating synthesis and bioavailability via IGF1-binding proteins [48], as well as amplification of signal transduction capacity [49,50,51]. Insulin and IGF-1 both transactivate each other’s receptors, as well as form cross hybridised receptors [52]. Chronically elevated IGF-1, and/or increased IGF-1 bioavailability and sensitivity, receptor expression, and amount of Ras protein prenylation [51] are strongly implicated in neoplasia and ageing [48,53,54,55,56], whilst IGF-1 knockdown within in vivo models show improved longevity [56,57].

In observational studies, low levels of insulin and IGF-1 have also been associated with reduced levels of pathologies. For example, elevated IGF-1 has been shown to correspond to a 69% increase in colorectal cancer risk, a 49% increase in prostate cancer risk, 65% increase in breast cancer risk, and a 106% increase in lung cancer risk [58] (relative risks). Notably, a recent meta-analysis involving over 30,000 participants indicated that IGF-1 within the range of 120–160 ng/mL was the optimum range associated with the lowest risk of all-cause mortality [58]. The participants in the present study fell well within this range during the P1 and P3 phase; however, during SuK (P2), IGF-1 significantly increased, which may confer an increased risk of all-cause mortality. Conversely, the lower levels of insulin and IGF-1 during the P1 and P3 phases may be of health benefit given that higher levels of IGF-1 and insulin are significant risk factors for various diseases.

Insulin/IGF-1 signalling inhibits FOXO activity via increasing phosphorylation, causing cytosolic sequestration, and suppressing BHB action on FOXO expression and nuclear translocation, through the Akt signalling pathway [59]. FOXO is a transcription factor which regulates the expression of a vast number of genes with functions associated with longevity, including cell cycle arrest, autophagy, and DNA damage repair [60], as well as regulating metabolism and antioxidant defence [61]. In addition to being a metabolic substrate, BHB also acts as a signalling molecule, modulating intracellular activity in cells across the body, such as regulating gene expression through inhibition of class I histone deacetylases (HDACs) via competitive inhibition [62]. Specifically, BHB prevents histone acetylation at the *FOXO* gene regions [18], and 12 h fasted mice have significantly increased levels of FOXO protein expression in the liver [63]. Based on our understanding of these cellular and intracellular signalling and fuelling dynamics, we propose that the low levels of insulin and IGF-1 maintained by the participants in our study during P1 and P2, along with BHB ≥ 0.5 mmol/L, through their lifestyle habits, are a logical and potentially effective way to slow and/or reduce cellular ageing. It is unlikely that life-long sustained vs. suppressed ketosis human trials will ever happen, and these indirect comparisons are our next best option, as we see in whole of life animal trials, maintaining minimal insulin demand and IGF-1 levels consistently results in optimum longevity [54,55,56,64].

Considering that our cohort is exclusively female, it is imperative to recognise, for global population health, the pressing importance of focusing on diabetes, hyperinsulinaemia (insulin may be inside reference ranges; however, chronic hypoketonaemia may indicate an individual’s hyperinsulinaemia threshold), obesity, and breast cancer. Given their widespread prevalence [65,66,67,68], understanding the intricate links between these conditions is critical in order to prevent occurrence and to improve outcomes. Our participant data show that long-term NK reduces fasting insulin, IGF-1, and glucose. This data adds to existing evidence that sustaining a lifestyle which promotes ketosis is an effective modality for the prevention and management of both type 1 and type 2 diabetes [69].

### 3.3. Thyroid—Free T3

Along with increased glucose and insulin concentrations, SuK (P2) resulted in increased levels of free T3 (fT3). Given fT3 is highly involved in the transcription and translation of OXPHOS proteins, it would be expected that being in ketosis would come with higher levels of fT3 than a suppressed ketosis state. Being in ketosis is highly dependent on OXPHOS capacity. We found, in our healthy long-standing-ketosis-maintaining cohort, that their fT3 was significantly lower than after 21 days of suppressed ketosis. A plausible explanation is that ketosis, a fasting-mimicking metabolic state, reduces thyroid hormone (TH) demand due to less ROS damage on OXPHOS proteins and mt IMM lipids, such as cardiolipin [70], and may increase sensitivity, such as increasing mitochondrial fT3 receptors and/or increasing monocarboxylate transporter 8 [71]. In addition, BHB has an epigenetic regulatory role of its own, enabling increased transcription of OXPHOS proteins [20]. Short durations, such as 21 days which, evolutionarily, would be akin to a short summer/autumn, are within the thyroid’s capacity to deal with. However, in possible similarity to the pancreatic beta cells, chronic demand of the thyroid to produce increased amounts of TH may result in mechanisms that downregulate either production or conversion of T4 to fT3, respectively.

In P2, our cohort were not chronic long-term hyperinsulinaemic. Hence, they had the earlier phase of greater demand of thyroid hormone (TH). If those 21 days turned into 21 years, it is arguable that, over that time frame, TH may become low, more specifically T3, in concordance with the research literature in T2DM hyperinsulinaemic populations. The aforementioned low T3 levels in our cohort when in ketosis, P1, and P3, could lead clinicians to mistakenly diagnose a metabolic phenotype 1 individual (see methods 2.2) with hypothyroidism. Therefore, it is worth highlighting that T3 levels were still within normative ranges; however, this information should assist clinicians and researchers by indicating a need for nuance and metabolic context when interpreting thyroid biomarkers.

### 3.4. OGTT

Our data indicate that the participants enrolled in this study were able to maintain normal glycaemic responses throughout the OGTT following each of the phases (Figure 5), highlighting that prolonged ketosis did not hinder metabolic flexibility. However, in the first 2 h of insulin response, there appears to be a shift to the right for P1 and P3. This is likely due to a reduced frequency and load from a large glucose bolus exposure, therefore increasing the time to peak for bolus insulin synthesis and secretion. Concurrently, plasma glucose appears to be greater during the first 2 h in these two phases, and higher peaks of glucose are also seen in P1 and P3 compared to the P2 phase (although these are not statistically significant). This pattern may be incorrectly labelled as a lack of sensitivity to insulin; on the contrary, this is the sum of the exogenous glucose from the OGTT, plus hepatic glucose output, which does not abate from the one-time glucose bolus.

The dosage of dietary glucose administered during the OGTT compounds with the hepatic glucose output, and therefore contributes to an elevated peak of glucose concentrations during P1 and P3 (Figure 4A). When the participants were fat fuelling (metabolic phenotype 1), their glucose needs were largely dependent on hepatic provision via gluconeogenesis and glycogenolysis. Sustained glycogenolysis during an OGTT is also seen in hyperinsulinaemic individuals (metabolic phenotype 3), where the liver is pathway-selective insulin-signalling-resistant, suppressing ketogenesis and inhibiting beta-oxidation whilst increasing de novo lipogenesis, and glycogenolysis is not inhibited [72]. The chronic hyperinsulinaemic state is also coupled with a higher glucagon state [73,74], adding to hepatic signalling that maintains hepatic glucose provision to the wider system.

Hypothetically, under an evolutionary context, selection pressure would have favoured the ability to adapt to and maintain NK due to seasonal food availability and intermittent CR/fasting, meaning the body’s glucose needs would have been met by hepatic gluconeogenesis and glycogenolysis. If an (infrequent) opportunity to consume a high carbohydrate load would have presented itself, the subsequent increased insulin secretion would likely not have inhibited gluconeogenesis and glycogenolysis during the one-time exposure, given that the body is adapted and reliant on hepatic glucose as its main glucose source [8,28]. If insulin, in this one instance, were to inhibit gluconeogenesis and glycogenolysis, whilst facilitating oral glucose load myocyte uptake, a potential case of hypoglycaemia with hypoketonaemia and inhibition of beta-oxidation may simultaneously occur, which would be potentially fatal. In this metabolic phenotype 1 context [8], an infrequent one-time bolus insulin secretion does not inhibit gluconeogenesis and glycogenolysis; this may incorrectly be interpreted as hepatic insulin resistance, as is the case for hyperinsulinaemic T2DM (stage-3 metabolic phenotype 3) individuals [8]. This has not been observed in our cohort, as ketogenesis declined during the first 2 h, indicating that the liver is being affected by the bolus insulin release and is selectively responding based on metabolic phenotype physiological state and adaptation. Like switches and gates, the metabolic phenotype signature changes hepatic responses to a bolus insulin signal.

Overall, our data indicates that long-term ketosis does not appear to negatively affect the insulin-dependent glucose uptake nor reduce carbohydrate tolerance. In fact, following SuK P2, participants demonstrated a significantly elevated peak level of insulin in response to a glucose challenge across the OGTT, compared to P1 and P3. Furthermore, in all phases, insulin levels were at their lowest and plateauing after 240 min; glucose was on a rise upwards to basal P2 fasting levels after 180 min in P2, whereas glucose was further declining back to basal fasting levels in P1 and P3. Together, these findings indicate a lower total insulin requirement to maintain lower glucose levels when in a state of NK, whereas SuK was associated with an increased insulin requirement. Maintaining lower–normal glucose levels with lower insulin and IGF-1 levels is associated with improved health outcomes, decreasing risk of insulin resistance and T2DM, reducing chronic diseases, and also improving longevity and healthy ageing [56,57]. This suggests that maintaining a long-term metabolic phenotype 1 profile may aid in maintaining a healthier healthspan and lifespan.

We previously hypothesised that the addition of the BHB sensitivity assay in an OGTT challenge would help to differentiate between different metabolic phenotypes with improved resolution [6,8]. Here we show that, indeed, the combination of insulin and BHB measurements throughout the OGTT helps to differentiate early stage hyperinsulinaemic individuals (metabolic phenotype 3, stage 1 or 2) or prior metabolically unwell individuals who have restricted carbohydrates and gone into ketosis (metabolic phenotype 4) from long-standing healthy-ketosis-living individuals (metabolic phenotype 1). The combination of glucose, BHB, and insulin response measurements help to provide greater resolution in understanding metabolic health and helps clinicians and researchers to better classify individuals when designing trials or analysing data.

During P2, the participants were exposed to an increased frequency (ad libitum, spread over three times a day SUK diet recommended, which prevents TRF-induced ketosis), dose (glycaemic load, SUK diet recommendation to consume at least 267 g of carbohydrate per day), and duration (21-day intervention) of dietary glucose, consequently repeatedly triggering bolus insulin release (equating to an equivalent carbohydrate exposure of approximately 63 OGTTs in 21 days). The increased bolus insulin secretion signals to the liver to temporarily reduce glycogenolysis; this is considered hepatic insulin sensitivity (response seen in metabolic phenotype 2 and not phenotype 3, stage 3), as hepatic glucose output is reduced in response to the insulin signal. We have come to consider this the normal and healthy response, which is likely correct for those consuming a ketosis-suppressive diet and who do not have any chronic ageing and hyperinsulinaemia disease. However, if we were to consider humans under an evolutionary context, with less frequency, dose, and duration of exogenous carbohydrate exposure, then it is arguable that what we see in our cohort’s response curves in P1 and P3 would be the normal/healthy physiological responses. Hepatic glucose provision under the context of being in sustained NK would not be inhibited by bolus insulin secretion, and therefore this is not a case of pathological insulin resistance which logically only would be the case under a chronic hyperinsulinaemia and not an acute context (one-time OGTT for a metabolic phenotype 1 individual maintaining NK as a lifestyle). High levels of BHB would not be observed in hyperinsulinaemic/T2DM/CVD individuals, and therefore analysis of BHB response during an OGTT and/or for several consecutive days before the evening meal is essential for resolving a T2D glycaemic response (metabolic phenotype 3 spectrum) and those on the hyperinsulinaemic spectrum from those in ketosis [8].

We found that during both ketosis phases (P1 and P3), BHB concentrations began to recover following the glucose challenge at 180 min, with an overall U-shaped curve. However, participants in P2 did not mirror this response pattern and sustained low levels of ketones before and after 180 min following the glucose challenge, with a flat line pattern. These data indicate that consumption of a carbohydrate diet that suppresses ketosis for 21 days results in limited de novo ketogenesis, even following a 12 h fasting period, indicating adaptive changes and likely downregulation of cellular ketogenesis enzymes and activities.

### 3.5. Liver Markers

#### 3.5.1. GGT

There were significant findings regarding the effects of ketosis suppression and subsequent return to ketosis on liver markers. It is recognised that GGT is a diagnostic marker for many diseases in humans, including a fatty liver, T2DM, MetS, and AD, which are typified by hyperinsulinaemia [75,76,77]. In our study, SuK (P2) resulted in a significant increase in GGT levels, an enzyme associated with oxidative stress, low-grade inflammation, and insulin resistance [78,79]. Furthermore, GGT participates in the direct generation of ROS via a glutathione (GSH)/transferrin system, where, in the presence of molecular oxygen and iron/copper ions from transferrin and in the presence of cysteinylglycine (a product of GGT/GSH reaction) results in a paradoxical generation of ROS. This results in increased free radical and oxidative damage to nucleic acids and protein and lipid peroxidation [80]. Our findings suggest that suppressing ketosis may impose some degree of oxidative stress and inflammation on the liver, leading to increased GGT levels. GGT levels returned to near-baseline levels after our participants discontinued suppressing ketosis, indicating that carbohydrate restriction is an effective tool in correcting the significant increases in GGT.

High levels of GGT have been found to be associated with increased risk of MetS and impaired fasting glucose [78,81,82]. Our participants’ GGT levels in ketosis (P1 and P3) and SuK (P2) were within standard reference ranges. However, a study in a large nonobese population of nondiabetics, n = 1309, showed that a moderate elevation in GGT within normal reference ranges is a strong risk marker predictor for T2DM, independent of visceral fat, obesity, and HOMA [83]. Unwin et al. later corroborated this finding in a primary healthcare setting. After restricting dietary carbohydrates in 67 individuals, with a minimum of 3 months adherence and an average follow-up of 13 months, they found the improvements in GGT (reduction) had no correlation to weight loss [77]. GGT has also been shown to be associated with cognitive decline prior to vascular dementia in longitudinal observations (n = 452, average 80 years of age) [84]. With an increasingly growing aged population, monitoring GGT may also provide an ability to detect and intervene earlier in dementia prevention.

#### 3.5.2. PAI-1

The antifibrinolytic plasminogen activator inhibitor-1 (PAI-1) is the primary inhibitor of plasminogen activators (PAs) via inhibition of tissue-type PA (tPA) and urokinase-type PA (uPA) that proteolytically cleave zymogen plasminogen to active plasmin [85]. Elevated PAI-1 levels propagate a prothrombotic state [85]. We found a significant increase in PAI-1 levels during P2 compared to P1. Insulin has been shown to stimulate the secretion of PAI-1 by adipocytes, and there is a strong positive correlation between hyperinsulinaemia and elevated PAI-1 [86,87,88]. However, although fat mass increased after SuK (P2), it also returned to baseline levels in the return to ketosis (P3), whilst PAI-1 also significantly increased after P2, yet trended back, but not significantly, to baseline after P3. With the loss of gained fat mass after P3, with only a trend back for PAI-1, this indicates that other mechanisms outside of adiposity were involved.

PAI-1 circulates in the plasma at low levels (5–50 ng/mL), and its main pool is in platelets (approximately 300 ng/mL) [89]. Platelet activation is increased via increased PI3K, Akt, and PKC intracellular signalling, all of which are increased by hyperinsulinaemia and more so when glucose uptake insulin resistance develops [90,91,92]. In 2016, CVD mortality accounted for about 17.8 million deaths worldwide, where ischemic heart disease (IHD) and stroke contributed to 87% [4]. Disseminated intravascular coagulopathy (DIC) and thrombosis cause blockages of the blood flow to either the heart or brain, resulting in insufficient blood supply as well as increased atherosclerosis [93]. These processes are strongly associated with increased levels of PAI-1. NK may provide an effective strategy to reduce risk of DIC and thrombosis.

Hyperinsulinaemia increases gene expression of and stabilises PAI-1. Semad et al. demonstrated insulin increases in PAI-1 gene expression through a different signalling pathway to insulin-mediated glucose transport. Indicating that in the hyperinsulinaemia insulin-resistant state, where glucose tolerance declines, signalling by hyperinsulinaemia to upregulate PAI-1 gene expression is unimpeded and regulated by a pathway that does not become insulin-resistant [30,86].

PAI-1 contributes to an inflammatory response via infiltration of immune cells, specifically macrophages, in adipose tissue [85]. Adipocytes are a source of PAI-1 [85,94]. Our participants returned to their baseline mass with a concurrent loss of fat mass, indicating that the non-significant trend of PAI-1 returning to baseline from P2 to P3 was not associated with the significant loss of fat mass. As such, we may also conclude that, in our participants, the surge in PAI-1 during P2 was not due to enhanced adiposity.

A state of chronic inflammation, typical of conditions like obesity, T2DM, and MetS, is distinguished by the augmented expression of inflammatory adipokines, such as IL-6 and TNF-α [95]. These adipokines are known to upregulate PAI-1 expression within adipose tissue [96]. In our study’s data, however, we did not identify any marked alterations in these inflammatory markers across the different phases. These observations may explain how the inflammatory cytokines, notably IL-6 and TNF-α, did not precipitate PAI-1 increase from P1 to P2, suggesting that this increase was not caused by a surge in these cytokines.

Elevated, dose-dependent levels of PAI-1 are pro-tumourigenic, pro-angiogenic, and anti-apoptotic [97,98]. PAI-1 is one of the most highly induced proteins in metastatic invasive tumours and the tumourigenesis process [85,99]. PAI-1 binds to the low-density lipoprotein receptor-related protein 1 (LRP1) receptor, activating intracellular signalling cascades and modulating cell migration, such as mast cells in gliomas [85,100]. PAI-1 is a highly reliable prognostic and biomarker in a host of cancers, including breast [101,102,103,104,105], bladder [106,107], colon [108], gliomas [100,109], ovarian [110,111,112], non-small cell lung cancer [113], and renal [114] cancers.

PAI-1 is seen to increase with age; furthermore, PAI-1 is a part of the senescence-associated secretory phenotype (SASP) paracrine signalling pathway, inducing the SASP profile in neighbouring cells, therefore acting as both a marker and maker of cellular ageing and ageing-related pathologies [85,115,116]. Our data suggests that reducing PAI-1 through adopting a ketogenic diet may have the potential to carry wide health benefits.

### 3.6. Cytokines

It is often posited that inflammation precedes hyperinsulinaemia; however, our data does not support this. Where we observed increases in insulin and glucose, at the end of P2, CRP, interleukins, and TNF-α remained unchanged with no increase in CRP nor interleukin cytokines or TNF-α, indicating that inflammation known to be caused by these molecules, was not mediating the increased insulin levels that suppress ketogenesis. There were, however, increases in growth factors VEGF, EGF, and MCP which are discussed below.

#### 3.6.1. VEGF and EGF

Following SuK (P2), we observed significant increases in VEGF and EGF compared to baseline ketosis (P1) (Table 6; Figure 6A,B). This indicates that being in a state of ketosis does not overstimulate the production of these growth factors and chemokines, whereas being in a state of carbohydrate metabolism promotes their production. The concentration of these growth factors then trended back towards the baseline values after 21 days of returning to ketosis (P3). However, the concentrations were not significantly different compared to P2. This indicates that the relatively short period of carbohydrate fuelling is a sufficient time to elevate the concentrations of these growth factors in a way that cannot be fully recovered in 21 days after returning to ketosis.

Pericytes are supportive cells which wrap around blood vessels, serving as multilineage progenitor cells, and are essential for the development of new blood vessels [117]. Insulin stimulates pericytes to increase their production of VEGF, which, in turn, stimulates endothelial cells to grow and proliferate, facilitating angiogenesis [118]. The lower levels of insulin in the NK phases likely account for the lower levels of VEGF. Insulin and IGF-1 have been shown to promote the upregulation of VEGF or EGF [119,120,121,122]. EGF signalling is one of the key pathways involved in tumour development [123,124]. Ketogenic metabolic therapy (KMT) may aid in reducing the expression of EGF and VEGF.

Considering that our cohort is female, it is imperative to recognise the links between hyperinsulinaemia, metabolic health, and breast cancer. Women with diabetes have been shown to exhibit poorer outcomes for breast cancer compared to their non-diabetic counterparts [66]. Consistent with these findings, in vitro research has shown that treating cancer cells, particularly breast and pancreatic, with high levels of glucose initiates molecular alterations such as phosphorylation of EGFR, which promotes their proliferation [125,126,127]. The implications of hyperglycaemia also extend to treatment outcomes, with heightened glucose levels during chemotherapy leading to increased chemoresistance in tumour cells [128]. Beyond direct cellular growth effects, the hyperglycaemic state appears to compromise the body’s innate anti-tumour defences, notably by inhibiting neutrophil mobilisation, thereby granting tumour cells an immunological escape route and enhancing their metastatic capabilities [129].

SuK (P2) involved routine 3x a day feeding containing around 267 g of carbohydrate, resulting in the occurrence of hyperglycaemic and hyperinsulinaemic excursions equivalent to 3 OGGTs per day for 21 days, totalling 63 OGTTs. These periodic increases in glucose and consequent bolus insulin would not be captured in a fasting glucose/insulin test. This likely contributed to the upregulation of VEGF, EGF, and PAI-1.

#### 3.6.2. MCP-1

Following SuK (P2), MCP-1 expression significantly increased, returning to baseline following P3 (Table 6; Figure 6C). MCP-1 is a chemokine (also termed CCL2) involved in the recruitment of monocytes and produced by a range of cell types including monocytes/macrophages, epithelial, adipocytes, endothelial, and smooth muscle cells [130], cells which express high levels of insulin receptors. Insulin has been shown to increase levels of MCP-1 in adipose tissue of both lean and obese individuals [131]. Thus, carbohydrate-rich diets that suppress ketosis, resulting in elevated insulin during P2, may help to explain the significantly increased levels of MCP-1.

There are multiple lines of evidence from both human and murine studies which suggest that MCP-1 appears to be a key player in insulin resistance. MCP-1-deficiency ameliorates insulin resistance in mice via downregulation of ERK and p38MAPK phosphorylation in the liver [132]. Moreover, MCP-1 has been shown to mediate skeletal muscle inflammation and localised insulin resistance in mouse muscle in T2DM models [133]. Thus, NK may not only assist with regulating glycaemic control in T2DM, reducing insulin demand and exposure [134], but may help to ameliorate further MCP-1-mediated insulin resistance, further reducing insulin demand.

The reduction in MCP-1 during the ketosis phases indicate one manner by which a ketogenic state may possess protective effects. Murine studies have shown that insulin can increase the expression of MCP-1 via adipocytes [135], and stimulation of adipose tissue with MCP-1 can also induce dedifferentiation, which may contribute to the pathologies observed in obesity, such as cancer cell dedifferentiation, which occurs in their malignant transformation [135]. Elevated levels of MCP-1 have also been indicated in the pathophysiology of many other diseases, including age-related macular degeneration [136], allergic asthma [137,138], COVID-19, and CVD [139].

Our cohort showed no significant changes in the interleukins, except for a decrease in IL-1b from P2 to P3 (Table 6). IL-1b is a potent pro-inflammatory cytokine which becomes upregulated in response to pathogens and also in chronic disease [140]. IL-1b is a cytokine mainly produced by activated monocytes/macrophages [141]. Elevation of IL-1b in P2 compared to P3 correlates with the increased expression of MCP-1 we observed in this phase. However, we did not see an increase in IL-1b after SuK P2 from P1; this may be because our participants were in an anti-inflammatory state (P1) that persisted during the early days of SuK, which may have buffered/slowed down any change during that time.

Given that VEGF, EGF, and MCP-1 are often elevated in many cancers [142,143,144,145], KMT may be an effective way to support the action of certain cancer therapies, along with using the glucose-ketone index (GKI) calculator to measure therapeutic efficacy in metabolic management of brain cancers and likely other cancers [146]. Furthermore, KMT may be an effective stand-alone therapy for cancer. There have, indeed, been various human studies indicating that a ketogenic diet is tolerable for individuals with cancer [147], but also effective in reducing tumour burden and symptomatic disease [148]. Tumour cells are not well adapted to metabolising ketones, but instead predominantly depend on glucose for fuelling [149]. Limiting glucose availability for tumours by adapting into ketosis may therefore create a metabolically unfavourable environment for tumour growth, whilst also reducing insulin and IGF-1′s growth and division stimulating signals [150]. The data presented here indicate that long-term ketosis is safe in healthy populations; well-designed clinical trials would elucidate the value of such an approach in cancer therapy.

## 4. Strengths and Limitations

Our study is the first investigating a non-athletic, healthy pre-menopausal female population living in a long-term (> 1 year, group average of 3.9 years) habitual ketosis lifestyle for more than 80% of their year. Our study was also culturally and ethnically diverse, whereby participants followed their own lifestyle and food preferences. However, they were controlled (via photos and daily dietary diary) for each meal throughout the day during the 9-week experimental period. Extensive nutritional instructions/guidance (e.g., reminders to follow the SUK dietary recommendations during the SuK phase) were also given to each participant.

Another strength of our study is participant adherence and compliance, as participants recorded and photographed daily capillary measurements (glucose and BHB), for a 6-month pre-trial (between 4–6 pm as a more rigorous threshold as opposed to morning overnight fasted measures) and throughout the experimental period (9 weeks) four times per day. The use of standardised procedures, including laboratory visit, blood sampling time, and testing measurements, is another strength of this study. The combination of anthropometric and metabolic indices as well as various biomarkers and OGTT assessments provides further knowledge of the underlying metabolic responses of hyperinsulinaemia, and subsequently on potential healthspan/lifespan. Finally, all our main results withstand a p-value correction, indicating this study was fully powered.

On the other hand, variations of findings between studies may occur due to the study population (e.g., females vs. males); female participants may respond differently to male cohorts due to hormonal changes. In addition to this, training status of the participants (trained vs. untrained) [151,152] or the duration of the study protocol may play a role [153]. Further work on metabolic health between different age groups (e.g., young vs. elderly population) and in healthy individuals in NK (as controls) vs. people with pathologies (i.e., cancer, T2D or elderly population with ageing-associated diseases, such as sarcopenia) in larger cohorts is also needed. Future investigations should also conduct in-depth profiling analysis using RNA sequencing, proteomics, and metabolomics in response to hyperinsulinaemia.

Being a study on a wide range of physiological biomarkers (with different intrinsic magnitudes of variance), it is expected to have a large variability of p-values. We would like to highlight that while only insulin and IGF were used to calculate the sample size, most biomarkers showed statistical significance clearly below our multiple comparisons corrected p-value. Therefore, the borderline statistically significant findings in the measured cytokines and growth factors should be interpreted within the context of likely statistically underpowered tests on highly variable biomarkers and warrant further investigation as their trends are in resonance with the protective metabolic and inflammatory patterns we observed and that have been documented elsewhere.

## 5. Translational Importance

Recently, it has been shown in a large cohort study that a diet with a high proportion of carbohydrates significantly increases the risk of CVD [154]. This study, coupled with the studies discussed above, indicates that higher levels of insulin and IGF-1 are associated with increased morbidity and mortality risk. The current SUK Eatwell guideline recommendation to consume at least 267 g of carbohydrates a day effectively suppressed ketosis to a degree that by the end of this 21-day intervention, our participants were waking up with undetectable ketones on a capillary meter, indicating that insulin demand, secretion, and exposure had been enough to downregulate ketogenesis to even prevent a return to ketosis after an overnight fast. We therefore propose that the reduced concentration of BHB, accompanied by higher concentrations of insulin and IGF-1, may confer increased risk of morbidity and mortality over time, potentially increasing biological ageing rate.

## 6. Materials and Methods

### 6.1. Ethical Approval

Ethical approval was obtained by the College of Liberal of Arts and Sciences Research Ethics Committee, University of Westminster, United Kingdom (ETH2122-0634). All procedures were conducted in accordance with the Declaration of Helsinki and UK legislation. Written informed consent was obtained from all participants prior to their participation.

### 6.2. Participants

Ten healthy, habitually keto-adapted (living ketosis lifestyle prior to starting trial, self-reported average of 3.85 years), pre-menopausal women [age, 32.30 years ± 8.97; body mass index (BMI), 20.52 ± 1.39] were recruited. Participants were not receiving hormonal birth control and were classified as “metabolic phenotype 1” as defined by capillary BHB (> 0.3 mmol/L) and low fasting insulin < 130 pmol/L, with normo-glycaemia [30]. Habitual ketosis was determined by once-daily capillary BHB measurements between 4–6 p.m., before the evening meal, for 6 months prior to commencement of the study. A summary of the participants’ characteristics at baseline is given in Table 1.

Exclusion criteria included smoking, taking any medication, and evidence of metabolic, immunological, or CVD. Participants were required to complete a medical history questionnaire to confirm that they were free from any of the above diseases.

### 6.3. Study Design

**Figure 7:**
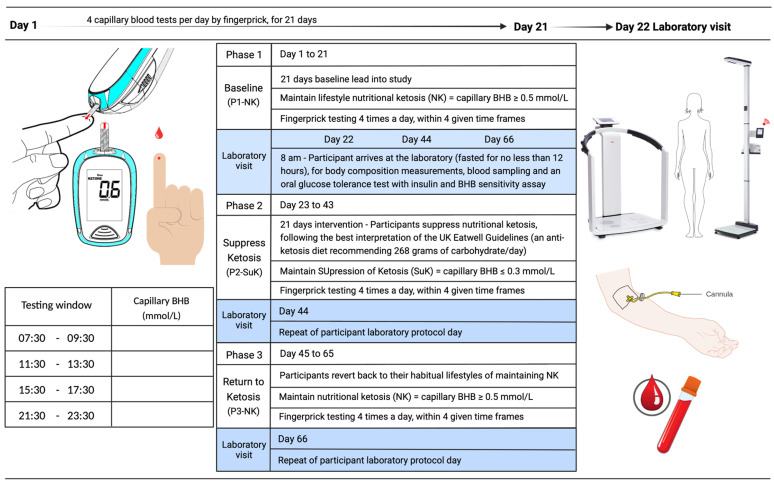
KetoSAge study design. Phase 1 and 3 covered the participants’ habitual ketosis lifestyle. Phase 2 was the interventional phase to suppress ketosis (SuK). Each phase was monitored via finger prick testing of capillary beta-hydroxybutyrate (BHB) concentration (mmol/L). Testing was conducted four times per day, prior to mealtimes at evenly spaced intervals. At the end of each phase, participants underwent laboratory testing for body composition and blood sampling for biomarkers associated with chronic diseases and ageing, and were given an oral glucose tolerance test (75g glucose in 250 mL water). Blood samples were taken at seven time points over 5 h. Whole blood glucose and BHB were measured sequentially in real time using the Keto-Mojo™ Meter, and plasma insulin sensitivity assay was conducted later using ELISA.

The study was an open-labelled, non-randomised cross-over trial with three phases: baseline nutritional ketosis (NK) (Phase 1; P1), suppression of ketosis (SuK) (Phase 2; P2) and removal of intervention, returning to NK (Phase 3; P3) (Figure 7).

For the duration of the study, participants were required to monitor their capillary glucose and ketone BHB concentrations (mmol/L) at four time points throughout the day to ascertain compliance (Table 2 and Table 3). These time points were between 7:30–9:30 a.m., 11:30–13:30 p.m., 15:30–17:30 p.m. and 21:30–23:30 p.m. Participants determined capillary glucose and BHB using a Keto-Mojo™ GKI multi-function meter (Keto-Mojo, Napa, CA, United States). This equipment was selected for its reliability and good diagnostic performance [155].

During P1, participants maintained lifestyle NK, as determined by maintenance of capillary blood concentration of BHB ≥ 0.5 mmol/L, through ad libitum consumption of a very-low-carbohydrate high-fat diet (VCHF), % carbohydrate:protein:fat = 8:17:75 [22,156] (this ratio is modulable according the metabolic health), ad libitum feeding within a time-restricted feeding (TRF) window, or a mixture of both (Table 2 and Table 3).

On day 22 (visit 1), participants attended the Human Physiology Laboratory at the University of Westminster at the same time of day (8 a.m.) in an overnight fasted state (> 12 h) for baseline testing. The baseline visit included anthropometric measurements, metabolic measurements, including exchange analysis (VO_2_, VCO_2_), venous blood sample, and an oral glucose tolerance test (OGTT) with BHB sensitivity. On day 23, participants suppressed ketosis (P2) and capillary BHB was targeted to be sustained at < 0.3 mmol/L for 21 days. Participants adapted out of ketosis during days 23 to 43 by following their healthiest interpretation (ad libitum) of the UK Eatwell Guidelines (% carbohydrate:protein:fat = 55:20:25), which recommend consuming at least 267 g of carbohydrate per day, divided over at least three meals.

On day 44 (visit 2), participants reported to the laboratory at 8 a.m. having fasted overnight to complete the same measurements as during visit 1. On day 45, the trial intervention was removed, and participants returned to their habitual lifestyle patterns, resulting in a return to NK (P3), and during days 45 to 65, they continued to monitor their capillary blood glucose and ketones, where BHB was maintained at ≥0.5 mmol/L, as in P1. On day 46 (visit 3), participants returned to the laboratory to repeat identical measurements as on previous visits. An overview of the study design is presented in Figure 7.

### 6.4. Anthropometric Measurements

Upon arrival at the laboratory, height (to nearest 0.1 cm) was measured using a stadiometer (Marsden HM-250P Leicester Height Measure), and body weight (to nearest 0.1 kg), BMI, fat mass, and total body water (TBW) were measured by bioelectrical impedance (BIA) using Seca^®^ (mBCA 514 Medical Body Composition Analyzer, Gmbh&Co. KG, Hamburg, Germany) with participants being 12 h fasted, with an empty bladder, and with standardised clothing. In addition, waist and hip circumference measures were obtained with a non-stretch anthropometric circumference measuring tape (Seca^®^ 201) while participants stood upright on both feet. The average value (cm) of three measurements was used for analysis.

### 6.5. Metabolic Measurements

Respiratory quotient (RQ) was measured by indirect calorimetry using a Quark RMR (COSMED srl, Rome, Italy) and was defined as the ratio of carbon dioxide (CO_2_) production to oxygen (O_2_) consumption. RQ was determined with the participants lying down at rest and with 15 min of lead time to allow respiration to equilibrate before measurements were taken. After RQ was determined, blood pressure was taken using an automatic upper arm blood pressure monitor (OMRON HEALTHCARE Co., Ltd., Kyoto, Japan).

### 6.6. Blood Collection

Following anthropometric measurements, a single-use sterile 22G Terumo (Japan, Tokyo) Versatus Winged and Ported IV Catheter (Cannula) was inserted into the participants’ antecubital vein for blood sampling. Saline solution flushes (0.9% NaCl, 5 mL, BD PosiFlush SP Syringe) were delivered in order to keep the intravenous line patent. A total of 2 mL of blood was drawn and discarded prior to each blood draw to prevent blood sampling saline dilution.

Blood was drawn into tubes anti-coagulated with either ethylenediaminetetraacetic acid (EDTA) or lithium heparin (BD, Oxford, UK), ready for analysis by SYNLAB (see Section 6.7). Blood was also drawn into serum SST™ II Advance tubes with thrombin rapid clot activator and separation gel (BD, Oxford, UK) and left for 30 min at room temperature. Serum tubes were then centrifuged (Hettich Zentrifugen, Universal 320 R, Tuttlingen, Germany) at 3857× *g* for 10 min at room temperature. Serum samples were either sent to SYNLAB for analysis or aliquoted into cryovial tubes under sterile conditions and stored at −80 °C for later analysis by Randox (see Section 6.7).

### 6.7. Blood Profiling Analysis

Following blood draw, the blood samples were immediately sent to SYNLAB Belgium (Alexander Fleming, 3–6220 Heppignies–Company No: 0453.111.546) to determine the concentrations of the following markers: insulin, insulin-like growth factor 1 (IGF-1), insulin-like growth factor binding protein 3 (IGFBP-3), C-reactive protein (CRP), gamma-glutamyl transferase (GGT), cortisol, plasminogen activator inhibitor-1 (PAI-1), total cholesterol, high density lipoprotein (HDL) cholesterol, low density lipoprotein (LDL) cholesterol, triglycerides, thyroid stimulating hormone (TSH), free triiodothyronine (T3), reverse T3, and thyroxine (T4).

At the end of the trial, frozen serum samples were sent to Randox Ireland (55 Diamond Road, Crumlin, Co. Antrim, BT29 4QY, company number: NI015738) to determine the concentrations of various cytokines and growth factors. These included: epithelial growth factor (EGF), vascular endothelial growth factor (VEGF), interferon-gamma (INF-**γ**), monocyte chemotactic protein (MCP-1), tumour necrosis factor-alpha (TNF-ɑ), interleukin (IL)-1a, IL-1b, IL-2, IL-4, IL-6, IL-8, and IL-10.

### 6.8. Oral Glucose Tolerance Test

Following anthropometric and metabolic measurements and blood sampling, participants were subjected to an OGTT. 75 g of glucose in 250 mL water (prepared fresh on each day) was consumed by participants within 5 min. Blood samples were then drawn into EDTA tubes via cannula at 7 time points: 0 min (before glucose bolus), 30, 60, 120, 180, 240, and 300 min. All samples were immediately spun at 3857× *g* for 10 min at 4 °C to obtain the plasma fraction. Plasma was aliquoted under sterile conditions and stored at −80 °C for later batch analysis. Plasma insulin concentrations were determined by Quantikine ELISA (R&D Systems), following the manufacturer’s instructions. Samples were thawed once and analysed in triplicate. Throughout the OGTT, at each time point, venous whole blood was used to measure glucose and BHB concentrations, measured in triplicate, using a Keto-Mojo™ GKI multi-function meter.

### 6.9. Statistical Analysis

All data was found to be normally distributed by the Shapiro–Wilk test, and therefore parametric analyses were conducted. Repeated measures one-way analysis of variance (ANOVA) was used to evaluate differences in various parameters between the three phases (baseline ketosis P1, suppression of ketosis P2, and return to ketosis P3), or across time points (0, 30, 60, 120, 180, 240, and 300 min) for OGTT on glucose, insulin, and BHB. Tukey’s HSD test was used for post hoc analysis to perform pairwise comparisons, and *p* values < 0.05 were considered statistically significant. Data are presented as mean ± standard deviation, unless otherwise stated. Statistical analysis was performed and all figures were generated in GraphPad Prism (v9; San Diego, CA, USA).

### 6.10. Sample Size Calculation

Sample size was calculated based on pilot feasibility data with 5 participants put through all 3 phases. We calculated sample size using changes in fasted insulin and IGF-1 concentrations. The sample size was estimated using G*Power (v3.1) with an alpha level of 0.05, a power (1-β) of 0.80, a medium effect size of f = 0.5, and a conservative intra-measurement correlation of 0.5. This analysis recommended a sample size of n = 9, that predicted to produce results with an effect size of 1.1 d_z_ for paired comparisons.

## 7. Conclusions

Evolutionary evidence suggests that ancestral populations were predominantly adapted to patterns of intermittent and time-restricted feeding, as opposed to continuous nutritional intake, rich in farinaceous and sucrose carbohydrates that stimulate bolus insulin secretion. The escalating prevalence of T2DM, obesity, CVD, AD, and cancer observed in populations adhering to multiple substantial carbohydrate-dominated meals in developed nations is a testament to this. Individuals maintaining long-standing habitual NK, when subjected to 21 days of consuming carbohydrate to suppress ketosis, followed with restricting carbohydrate, reverted to an evolutionary ketotic state within one day, indicate metabolic flexibility and health. The negative changes in biomarkers associated with chronic diseases and ageing, which occur from a one-time excursion in a 1-year period of 21 consecutive days of suppressing ketosis, are rapidly restored after restoring the baseline dietary lifestyle of carbohydrate restriction which does not overstimulate insulin demand and secretion. Our data show that long-standing NK appears to provide major health benefits in the maintenance of euglycaemia, with low insulin and IGF-1, the triad of markers most strongly associated with chronic diseases and biological ageing. NK serves as a reliable surrogate marker for these parameters to understand an individual’s metabolic phenotype, and therefore risk. This study was conducted to establish a detailed metabolic phenotype biomarker profile in a long-standing healthy ketosis cohort, providing a NK control group for other studies to establish metabolic phenotypes in people with cancer, CVD, AD, T2DM, and ageing, and to assess treatment efficacy using KMT in gaining better health. Overall, sustained NK may mitigate hyperinsulinemia without impairing metabolic flexibility and carbohydrate tolerance in metabolically healthy individuals. Maintaining low insulin requirement and IGF-1 levels through endogenous NK may offer lower chronic disease risk, resulting in benefits to both lifespan and healthspan.

## Figures and Tables

**Table 1:** Participants’ characteristics. Measurements were taken following each of the study phases: baseline nutritional ketosis (NK) P1; intervention to suppress ketosis (SuK) P2; and removal of SuK returning to NK P3. Measurements were taken at 8 a.m. after a 12 h overnight fast; (n = 10).

	P1	P2	P3	ANOVA *p* Value	P1 vs. P2	P2 vs. P3	P1 vs. P3
**Age (years)**	32.30 (±8.97)						
**Height (cm)**	160.95 (±7.28)						
**Weight (kg)**	52.99 (±4.24)	55.65 (±4.10)	53.93 (±4.04)	<0.0001	0.0002	<0.0001	0.7888
**BMI**	20.52 (±1.39)	21.54 (±1.30)	20.82 (±1.46)	<0.0001	<0.0001	0.0025	0.0197
**Waist/Hip**	0.75 (±0.03)	0.77 (±0.03)	0.74 (±0.03)	<0.0001	0.0015	<0.0001	0.5361
**Waist/Height**	0.43 (±0.03)	0.45 (±0.03)	0.43 (±0.03)	<0.0001	0.0009	<0.0001	>0.9999
**Fat mass (kg)**	14.21 (±2.55)	15.88 (±2.23)	14.78 (±2.20)	<0.0001	0.0008	0.0057	0.1016
**TBW (L)**	28.15 (±2.87)	29.15 (±2.96)	28.42 (±3.15)	0.0005	0.0016	0.0262	0.3473
**RQ**	0.66 (±0.05)	0.72 (±0.06)	0.65 (±0.06)	0.0096	0.0427	0.0005	0.8606
**Systole (mmHg)**	103.25 (±6.24)	103.70 (±10.17)	100.00 (±9.54)	0.1455	0.9753	0.1746	0.2274
**Diastole (mmHg)**	70.75 (±4.91)	69.45 (±7.14)	68.15 (±7.36)	0.3227	0.8044	0.7147	0.1715

**Table 2:** Summary of fulfilled capillary BHB testing for all study participants across all phases (P1–P3). Measurements were taken following each of the study phases: baseline nutritional ketosis (NK) P1; intervention to suppress ketosis (SuK) P2; and removal of SuK returning to NK P3; (n = 10).

			Mean Capillary BHB Concentration (mmol/L)		
Participant	No of Tests Taken	% of Tests Fulfilled Out of 252	P1	P2	P3
**1011**	251	99.6	2.7	0.1	2.3
**1021**	252	100	2.8	0.1	2.2
**1031**	252	100	2.6	0.1	1.8
**1041**	252	100	1.5	0.2	1.6
**1051**	251	99.6	1.7	0	1.6
**1061**	245	97.22	0.7	0.1	0.8
**1071**	248	98.41	1.7	0.2	2.4
**1081**	250	99.21	2	0.1	1.2
**1091**	251	99.6	1.8	0.1	2.5
**1101**	252	100	1.5	0.1	2.4
**Mean**	250.4	99.37	1.9	0.1	1.9
**±SD**	2.15	0.85	0.7	0.1	0.6

**Table 3:** Percentages of capillary BHB readings as categorised by different cut-offs across the study phases.

	Capillary BHB (mmol/L)											
	≥ 0.5			> 0.3			≤ 0.3			< 0.1		
Participant	P1	P2	P3	P1	P2	P3	P1	P2	P3	P1	P2	P3
**1011**	100.00	2.38	95.18	100.00	4.76	98.80	0.00	95.24	1.20	0.00	28.57	0.00
**1021**	100.00	2.38	88.10	100.00	2.38	94.05	0.00	97.62	5.95	0.00	60.71	0.00
**1031**	100.00	2.38	92.86	100.00	2.38	95.24	0.00	97.62	4.76	0.00	59.52	0.00
**1041**	98.81	0.00	100.00	100.00	8.33	100.00	0.00	91.67	0.00	0.00	13.10	0.00
**1051**	100.00	0.00	97.62	100.00	1.19	98.81	0.00	98.81	0.00	0.00	94.05	0.00
**1061**	94.05	0.00	82.93	97.62	0.00	90.24	2.38	100.00	9.76	0.00	37.97	0.00
**1071**	96.30	4.82	97.62	97.53	4.82	97.62	2.47	95.18	2.38	0.00	1.20	0.00
**1081**	96.39	0.00	90.48	98.80	1.20	95.24	1.20	98.80	4.76	0.00	33.73	0.00
**1091**	98.81	0.00	98.80	100.00	1.19	100.00	0.00	98.81	0.00	0.00	21.43	0.00
**1101**	96.43	0.00	98.81	100.00	0.00	100.00	0.00	100.00	0.00	0.00	54.76	0.00

**Table 4:** Fasted insulin, IGF-1, glucose, and BHB across all phases. Measurements were taken following each of the study phases: baseline nutritional ketosis (NK) P1; intervention to suppress ketosis (SuK) P2; and removal of SuK returning to NK P3. Measurements were taken at 8 a.m. after a 12 h overnight fast; (n = 10; ^†^ n = 5).

	P1	P2	P3	ANOVA *p* Value	P1 vs. P2	P2 vs. P3	P1 vs. P3
**Insulin (pmol/L)**	33.60 (± 8.63)	59.80 (± 14.69)	31.60 (± 9.38)	<0.0001	0.0002	<0.0001	0.5361
**IGF-1 (µg/L)**	149.30 (± 32.96)	273.40 (± 85.66)	136.90 (± 39.60)	0.0015	0.0045	0.0055	0.4124
**Glucose (mmol/L)**	4.36 (± 0.53)	5.12 (± 0.59)	4.41 (± 0.30)	0.0015	0.0088	0.0177	0.9469
**BHB (mmol/L)**	2.43 (± 1.28)	0.18 (± 0.13)	2.31 (± 0.71)	0.0001	0.0012	<0.0001	0.9854
**IGFBP-3 (mg/mL)**	3.69 (± 0.56)	4.41 (± 1.27)	3.67 (± 0.70)	0.2357	0.3621	0.4272	0.9361
**IGF-1/IGFBP-3** **^†^**	0.14 (± 0.03)	0.25 (± 0.08)	0.15 (± 0.04)	0.0584	0.0870	0.1554	0.9049
**TSH (mU/L)**	1.40 (± 0.74)	1.56 (± 0.75)	1.25 (± 0.81)	0.3065	0.2334	0.4498	0.7742
**Free T3 (pmol/L)**	3.81 (± 0.28)	5.51 (± 0.72)	4.05 (± 0.54)	<0.0001	<0.0001	0.0015	0.3040
**Reverse T3 (nmol/L)**	0.29 (± 0.09)	0.26 (± 0.10)	0.25 (± 0.09)	0.6039	0.7030	0.9674	0.6323
**T4 (pmol/L)**	13.51 (± 1.61)	13.24 (± 1.49)	12.65 (± 0.66)	0.2125	0.8795	0.3059	0.2099

**Table 5:** Concentrations of fasted liver markers measured across all phases. Measurements were taken following each of the study phases: baseline nutritional ketosis (NK) P1; intervention to suppress ketosis (SuK) P2; and removal of SuK returning to NK, P3. Measurements were taken at 8 a.m. after a 12 h overnight fast; (n = 10; ^†^ P3, n = 9; ^§^ P1, P2, P3, n = 5).

	P1	P2	P3	ANOVA *p* Value	P1 vs. P2	P2 vs. P3	P1 vs. P3
**Triglycerides** **(mg/dL)**	66.80 (± 28.00)	66.10 (± 21.09)	79.30 (± 45.88)	0.5018	0.9972	0.6629	0.6270
**Total cholesterol (mg/dL)**	231.50 (± 62.42)	188.50 (± 30.28)	210.20 (± 43.44)	0.0335	0.0802	0.2132	0.1061
**HDL cholesterol (mg/dL)**	70.10 (± 10.37)	72.70 (± 13.59)	69.80 (± 11.84)	0.6231	0.7460	0.6762	0.9943
**LDL cholesterol (mg/dL)** ^†^	4.46 (± 2.03)	3.13 (± 0.91)	3.96 (± 1.34)	0.0888	0.1798	0.3280	0.1498
**Triglycerides/HDL** **(mmol/L)**	1.01 (± 0.55)	0.95(± 0.38)	1.25 (± 0.90)	0.3804	0.9478	0.5358	0.5515
**CRP (Ultra-Sensitive) (mg/L)** ^§^	1.00 (± 1.19)	1.16 (± 1.56)	1.35 (± 2.23)	0.7103	0.9938	0.7477	0.7728
**Gamma-GT (U/L)**	9.60 (± 3.13)	12.40 (± 2.55)	9.70 (± 2.50)	0.0029	0.0087	0.0286	0.9885
**Cortisol (µg/dL)**	12.62 (± 5.27)	11.27 (± 5.85)	13.19 (± 5.22)	0.3574	0.6886	0.4087	0.8258
**PAI-1 (ng/mL)**	13.34 (± 6.85)	16.69 (± 6.26)	17.05 (± 5.58)	0.0431	0.0428	0.9483	0.1373

**Table 6:** Concentrations of fasted growth factors and cytokines across the different phases of the study Measurements were taken following each of the study phases: baseline nutritional ketosis (NK) P1; intervention to suppress ketosis (SuK) P2; and removal of SuK returning to NK, P3. Measurements were taken at 8 a.m. after a 12 h overnight fast; (P1, P2, n = 10; P3, n = 9).

	P1	P2	P3	ANOVA *p* Value	P1 vs. P2	P2 vs. P3	P1 vs. P3
**EGF (pg/mL)**	33.02 (± 30.96)	50.13 (± 38.19)	37.82 (± 26.81)	0.0139	0.0450	0.3473	0.0478
**VEGF (pg/mL)**	93.93 (± 54.30)	147.33 (± 100.03)	134.80 (± 98.79)	0.0147	0.0314	0.2102	0.0801
**Interferon-γ** **(pg/mL)**	1.14 (± 2.64)	0.72 (± 1.05)	0.57 (± 0.90)	0.3755	0.7019	0.2452	0.6019
**(MCP-1) (pg/mL)**	103.98 (± 39.30)	192.53 (± 84.73)	128.52 (± 51.80)	0.0026	0.0137	0.0175	0.2622
**TNF-α (pg/mL)**	2.23 (± 1.75)	2.66 (± 1.26)	2.09 (± 0.97)	0.1387	0.3887	0.0785	0.8430
**IL-1a (pg/mL)**	0.30 (± 0.40)	0.26 (± 0.25)	0.26 (± 0.25)	0.3230	0.6266	0.5406	0.5104
**IL-1b (pg/mL)**	2.23 (± 3.42)	1.85 (± 2.02)	1.71 (± 2.04)	0.3090	0.7045	0.0381	0.4989
**IL-2 (pg/mL)**	1.92 (± 1.48)	1.71 (± 1.16)	1.94 (± 1.37)	0.2932	0.4409	0.7569	0.3809
**IL-4 (pg/mL)**	2.14 (± 0.80)	2.06 (± 0.99)	2.25 (± 1.17)	0.4635	0.5358	0.5138	0.9090
**IL-6 (pg/mL)**	0.95 (± 0.80)	1.22 (± 1.11)	0.84 (± 0.56)	0.5034	0.9238	0.5677	0.5771
**IL-8 (pg/mL)**	8.91 (± 9.56)	8.60 (± 5.93)	8.08 (± 6.30)	0.6738	0.9966	0.5725	0.8009
**IL-10 (pg/mL)**	0.61 (± 0.37)	0.68 (± 0.46)	0.53 (± 0.25)	0.4323	0.9084	0.4420	0.5573

## Data Availability

The data presented in this study are available from the corresponding author upon reasonable request.
